# Sex Differences in Medication and Primary Healthcare Use before and after Spousal Bereavement at Older Ages in Denmark: Nationwide Register Study of over 6000 Bereavements

**DOI:** 10.4061/2011/678289

**Published:** 2011-08-10

**Authors:** Anna Oksuzyan, Rune Jacobsen, Karen Glaser, Cecilia Tomassini, James W. Vaupel, Kaare Christensen

**Affiliations:** ^1^The Danish Aging Research Center, Institute of Public Health, University of Southern Denmark, J. B. Winsloews Vej 9B, 5000 Odense, Denmark; ^2^Laboratory of Survival and Longevity, Max Planck Institute for Demographic Research, Konrad-Zuse Street 1, 18057 Rostock, Germany; ^3^Institute of Gerontology, King's College London, Melbourne House, Strand, London WC2R 2LS, UK; ^4^Department SEGES, University of Molise, Via F. De Sanctis, 86100 Campobasso, Italy; ^5^Population Studies Department, London School of Hygiene and Tropical Medicine, Keppel Street, London WC1E 7HT, UK; ^6^The Danish Twin Registry, University of Southern Denmark, J. B. Winsloews Vej 9, 5000 Odense, Denmark; ^7^Department of Clinical Biochemistry and Pharmacology and Department of Clinical Genetics, Odense University Hospital, Soender Boulevard 29, 5000 Odense, Denmark

## Abstract

*Background*. The study aimed to examine sex differences in healthcare use before and after widowhood to investigate whether reduced healthcare use among widowers compared with widows may partially explain excess mortality and more adverse health outcomes among men than women after spousal loss. *Methods*. All individuals alive and aged at least 60 years in 1996 and who became widowed in the period from 1996 to 2003 were selected from the 5% sample of the total Danish population and all Danish twins. The healthcare use was assessed as the average daily all-cause and major system-specific medication use and the average annual number of visits to general physicians (GPs). *Results*. The average daily use of all-cause and major system-specific medications, as well as the number of GP visits increased over the period from 1 year before and up to 5 years after a spouse's death, but there were no sex-specific patterns in the trajectories of medication use and number of GP visits after conjugal loss. *Conclusion*. We found little support for the hypothesis that reduced healthcare use contributes to the explanation of more adverse health outcomes after conjugal loss in men compared with women in Denmark.

## 1. Introduction

There is a mounting research literature showing a mortality and health disadvantage after spousal bereavement [1]. Elevated risks of death after conjugal loss among middle- and old-aged persons were found for all-cause mortality and most major causes of death, such as cancers, cardiovascular diseases (CVD), respiratory diseases, accidents, and violence in Finland, UK, and the US [2–5]. This excess mortality among the bereaved usually occurs within the first six months following the death of a spouse [3, 4]; however, some studies have suggested that it remains high many years after bereavement [6, 7]. In addition to mortality, the loss of a spouse was found to be associated with higher levels of depressive symptoms, poorer physical and cognitive function, worse self-rated health, and an increased risk of institutionalization in the US, Chinese, and Finish middle- and old-aged surviving spouses [8–12]. The adverse effects of spousal loss on health and mortality were independent of socioeconomic and health characteristics [8, 10, 13].

Previous research has shown significant sex differences in the widowhood effects: greater adverse effects on mortality and health have been found among widowers [1, 3, 9, 10, 14, 15]. Nevertheless, a few studies reported excess all-cause mortality after spousal loss to be very similar in men and women [6, 16]. There is also conflicting evidence regarding sex differences in the effects of bereavement on depressive symptoms and other dimensions of mental health [12, 14, 17]. 

Although the literature on widowhood effects on health and mortality in Denmark is limited, available data suggest that, as in the predominantly North American literature, the excess mortality is substantially higher among middle-aged and elderly widowers than among the same-aged widows [7, 18, 19]. A register study among the total Danish population aged 50 and over found that the oldest-old widowers had substantially higher suicide risks than the same-aged widows, but the sex differences were less pronounced in the younger age groups [7]. However, another register-based study in Denmark among 25–60 years old individuals revealed that the relative risk of suicide and risk of death from other causes after conjugal loss was substantially higher in men than in women. A study based on the total Danish twin population aged 50 to 70 indicated higher hazard rates after the death of a spouse and co-twin among men than among women, although the timing patterns were similar in both sexes [19]. Evidence for sex differences in widowhood effects on cancer incidence and cancer mortality in other Scandinavian countries is inconclusive [20, 21]. Although cancer incidence changed in the same direction among Swedish widowers and widows, the risk of esophageal, lung, and all cancers was higher in men than in women, whereas the risk of pancreatic cancer significantly increased and the risk of melanoma, skin, cancer and non-Hodgkin's disease significantly decreased in women only [20].

Several explanations have been proposed for gender differences in health and morality following bereavement. First, as most studies have generally demonstrated greater longevity and health benefits for married men than for married women, it has been suggested that conjugal loss may be more stressful for widowers compared with widows [15]. However, it is still unclear why physiological changes underlying reactivity to stressful life events should differ by sex. Second, it has been suggested that the adverse effects of widowhood on health and mortality may be due to the loss of social, material, and task support [3, 15]. Finally, there has been some investigation of the impact of losing a spouse on health behaviors, such as smoking, alcohol consumption, diet, and interaction with healthcare services [22–26]. 

Despite growing research on the adverse widowhood effects on health and mortality, existing studies that examined sex differences in lifestyle behaviors and social assistance as explanations for sex differential effect of widowhood on health and mortality had small sample size [23, 25, 27, 28], short follow-up period [29], excluded old-aged individuals [24], had no information on nonrespondents [9], and were based on cross-sectional data [30]. Studies on the changes in health maintaining behavior around the time of widowhood as underlying mechanism for adverse bereavement effect on health and mortality are scarce [26, 31].

The present study aimed to investigate sex differences in short- and longer-term healthcare use following conjugal loss by comparing medication and primary healthcare use before and after a spouse's death. We hypothesized that after a spouse's death elderly men will reduce medication use and visits to general practitioners (GPs), while women will maintain or increase their healthcare utilization compared to their preloss level. If so, such reduction in medication use and number of GP visits among men may contribute to the explanation of more adverse health outcomes after conjugal loss among men than women.

## 2. Materials and Methods

In April 1968, the Civil Registration System (CRS) was introduced in Denmark, where each resident is assigned the 10-digit unique personal identifier, the Central Personal Register (CPR) number. The CPR-number was used to link the diverse computerized registers covering the total Danish population: Danish Demographic Database (DDB) (demographics, residence, migration, and civil status since 1968), the Prescription Medicine Register (medicine-related information since 1995), the Health Insurance Register (information on primary care services since 1997), and the Danish Twins Register (demographics, residency, zygosity). Prior studies have proved the register data in Denmark to be reliable and valid with no linkage problems among registers [32–34].

The study is based on the 5% sample of the total Danish population and all Danish twins identified through the CRS and the Danish Twin Register, respectively. The total twin population was included to increase the sample size of older people, especially men, who became widowed in the period from January 1, 1996 to January 31, 2003. Previous research in Denmark demonstrated that twins are representative of general populations in terms of health trajectories, psychological function, and all-cause and cardiovascular mortality and, thus, are good population models for epidemiological and demographic research [35, 36]. Additionally, the analysis of age-specific death rates in the twin and 5% population by marital status in 1998 and 2000 showed patterns similar to that in the total Danish population (data not shown).

All individuals alive and aged at least 60 years by January 1, 1996 were initially selected. Further we selected all individuals who became widowed in the period from January 1, 1996 to January 31, 2003. This observation period was chosen as the Prescription Medicine Register is only available from 1995 onwards, and the updated civil status was available until January 31, 2003. Furthermore, the analysis of primary healthcare use was restricted to the period from January 1, 1997 to January 31, 2003 because the Health Insurance Register only began in 1997. Survival status was available through December 2006.

The Prescription Medicine Register contains detailed Anatomical Therapeutic Chemical classification system (ATC) codes of prescribed medications and their sublevels, dates of purchase, daily defined dose (DDD), and other drug-related information. The defined daily dose of a medication is based on the accepted average dose per 24 hours for that medicinal product facilitating comparison of consumption across different medications [37]. All-cause medication use was estimated within 1 year before and up to 5 years after spousal death in the average daily defined dose (DDD) by dividing the total DDDs dispensed for all medications combined within 1 year to 365.25 days. If a surviving spouse died within a follow-up year, the average DDD was calculated by dividing the total DDD dispensed during that follow-up year to the number of days survived. Because previous studies showed sex differences in cause-specific medication use, the medication use was also assessed for several major system-specific medications (the anatomical main group level 1): cardiovascular (ATC-C), nervous (ATC-N), respiratory (ATC-R), and alimentary tract and metabolism (ATC-A) medications.

The use of primary healthcare was assessed as the average number of visits to GPs per year within 1 year before and up to 4 years after spouse's death. Similar to the estimation of medication use, the average number of GP visits in the follow-up years was adjusted to the time at risk. Additional analyses of all-cause and system-specific medication use before and after widowhood among individuals conditional on their survival to the third and fifth follow-up years were conducted to investigate whether selective dropout due to death has an effect on the trajectories of healthcare use. The age at widowhood was categorized into three groups to avoid small number of users in each age group: 60–69, 70–79, and 80+ years. Data analysis was performed using Intercooled Stata 10.0. Chi-square tests were used to examine sex differences in the prevalence of medication use and two independent sample *t*-tests were used to examine sex differences in the average medication use and number of GP visits 1 year before and 1 year after widowhood and the sex gap in the change of healthcare use over the two time points. 

## 3. Results

In total, there were 6421 individuals (66.5% women) aged at least 60 by January, 1996 and became widowed during the observation period from January 1, 1996 to January 31, 2003.  Table 1 presents the percentage of men and women taking prescription medications 1 year before and 1 year after widowhood. In general, all-cause and system-specific medication use 1 year before and 1 year after a spouse's death was more frequent among women than men (Table 1). However, there were almost no gender differences in the prevalence of ATC-A medications either one year before or after widowhood (Table 1). 

Table 2 shows the average DDD for ATC medications dispensed 1 year before and after widowhood and the mean difference in the average DDD between the two time periods. Generally, the highest average DDD was found for ATC-C medications, followed by ATC-N and ATC-R medicines in both men and women. Women had higher average DDDs for all-cause and ATC-N medications compared with men in both the year before and after widowhood. Men, generally, had higher average DDDs for ATC-R medications, although sex differences were not always statistically significant. The sex-specific patterns for ATC-C and ATC-A medications were less clear (Table 2). As expected, at each cross-sectional time point, the average DDD was higher in older age groups.

The average DDD for all-cause and systems-specific ATC medications increased from 1 year before to 1 year after widowhood for both men and women (Table 2). The highest increase in average DDD was observed in the ATC-C and ATC-N medication use, followed by ATC-R and ATC-A medications. The increase in all-cause, ATC-C, and ATC-R medication use was slightly higher in men. However, the sex difference was statistically significant in the oldest age group for all-cause (*P*-value = 0.02), ATC-C (*P*-value = 0.04), and ATC-A (*P* = 0.02) medications. 

**Figure 1:**
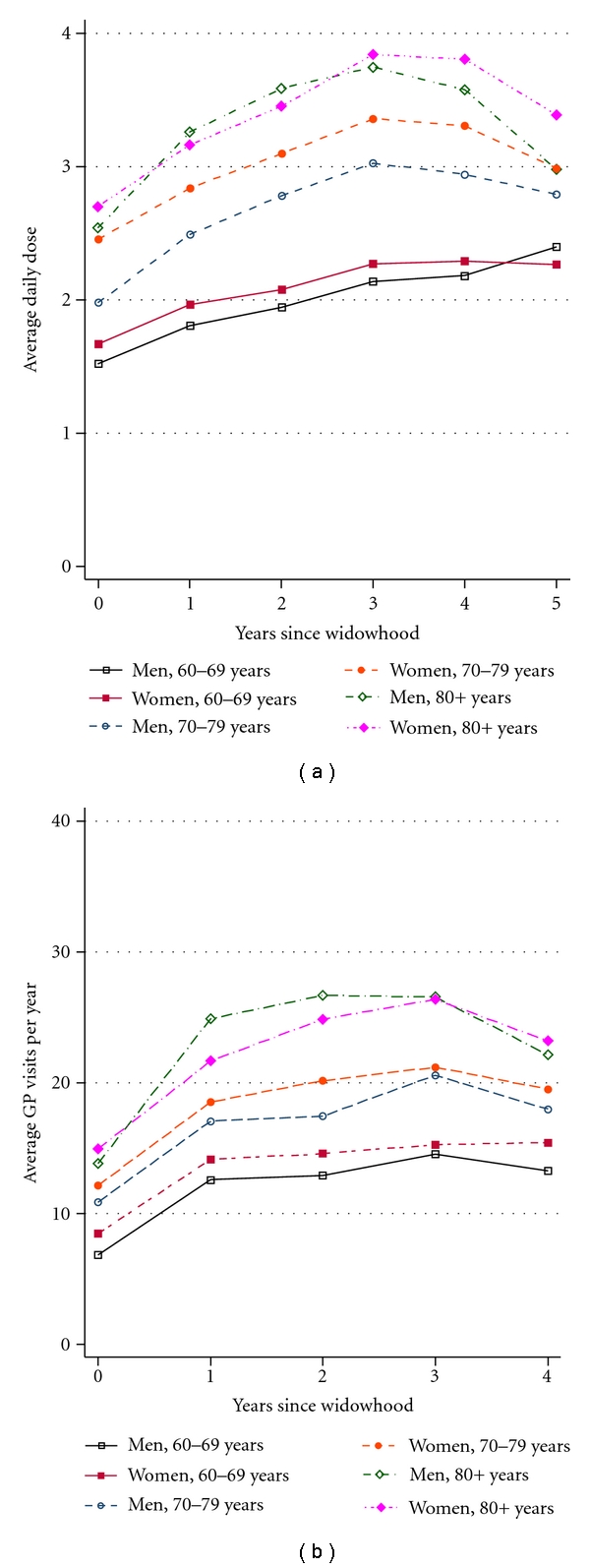
All-causes medication use and number of GP visits before and after widowhood.

**Figure 2:**
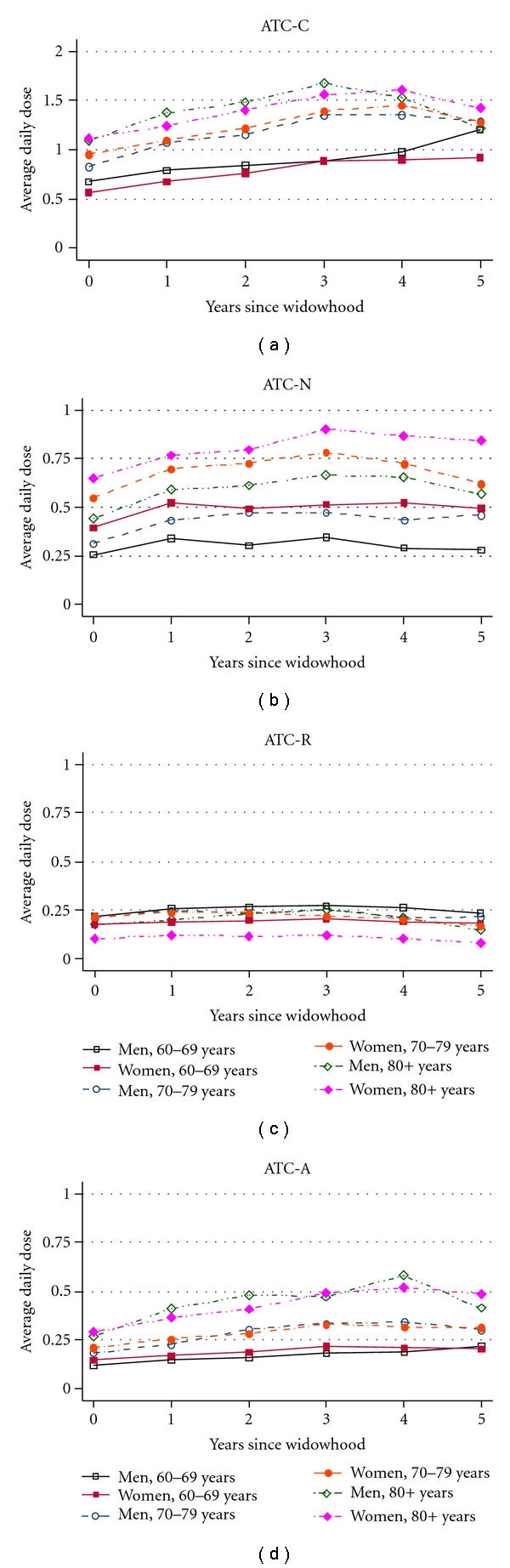
System-specific medication use before and after widowhood.

 Further analysis was done to examine whether there was a sex-specific pattern in all-cause and system-specific medication use up to 5 years after conjugal loss. The average DDD for all-cause medications increased between the year before and the third or fourth year following widowhood and remained almost constant or slightly decreased afterwards (Figure 1). Similar patterns were indicated for system-specific medication use, although with much smaller variations over time (Figure 2). However, there was no sex-specific pattern in the change of all-cause and systems-specific medication use over 1 year before and up to 5 years after widowhood. Among system-specific medications, the most pronounced changes were observed for ATC-C agents, except for the 60–69 years old age group, followed by ATC-N and ATC-A medications. The increase in the ATC-N medication use seems to be steeper within the first year after widowhood, but the pattern was similar in both sexes and all age groups. The smallest changes over the follow-up period were indicated for ATC-R medication use. 

Analogous findings were obtained when the average number of GP visits per year was considered as an indicator of healthcare utilization (Figure 1). Women had slightly more GP visits in all age groups compared with men. The average number of visits increased with advancing age, but these changes were similar in men and women. Similar patterns were indicated when the median number of GP visits was considered. 

**Figure 3:**
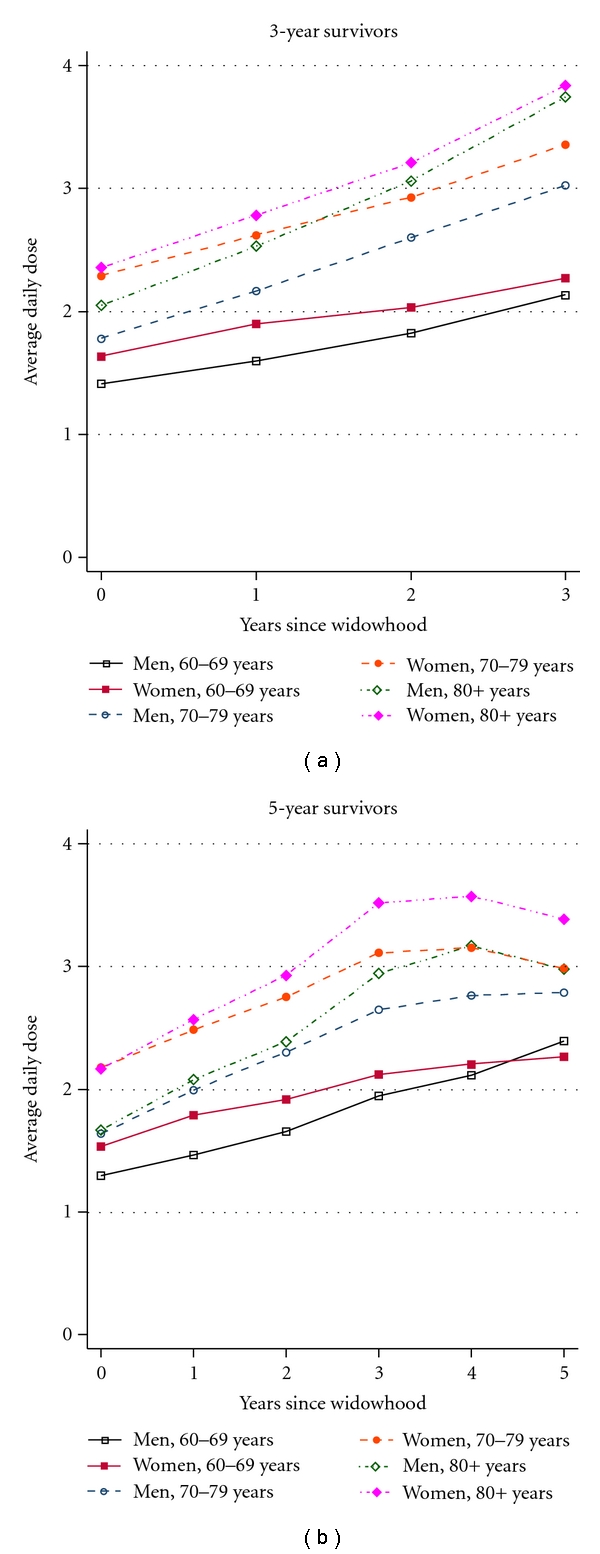
All-cause medication use among 3- and 5-years survivors.

To investigate whether selective dropout due to death has an effect on the trajectories of healthcare use, we analyzed all-cause and system-specific medication use and number of GP visits before and after widowhood among individuals conditional on their survival to the third and fifth follow-up years. The trajectories for all-cause (Figure 3) and system-specific medication use remained unchanged when the analysis was restricted to three- and five-year survivors after spousal loss. Also, these results remained unaltered when the change in medication use 1 year before and after widowhood was evaluated among users only or when medication use was estimated for several therapeutic subgroups (e.g., psycholeptics, psychoanaleptics, cardiac therapy, diuretics, beta blocking agents, calcium channels blockers, and agents acting on rennin-angiotensin system) (data available on request).

## 4. Discussion

The present study aimed at comparing medication and primary healthcare use around the time of widowhood among men versus same-aged women. It showed that all-cause and major medication use, as well as primary healthcare use, increased shortly after conjugal loss, as well as in the 3-4 years following spousal bereavement, and remained constant or slightly decreased thereafter. The trajectories of all-cause and system-specific medication use and the annual number of GP visits over the period from 1 year before and up to 5 years after conjugal loss were similar in men and women. Therefore, we found little support for the hypothesis that widowers reduce medication use or visits to GPs, while widows utilize healthcare services to a degree similar to that of a preloss level. The increasing number of GP visits and all-cause and system-specific medication use is likely to reflect the effect of age on the healthcare use rather than the effect of spousal loss. 

Compared to the growing research on widowhood-mortality association, the number of studies that investigated the underlying mechanisms responsible for poor outcomes following the death of a spouse, and, in particular, the reasons why widowers fare worse than widows is less extensive. Thompson and colleagues revealed that although within 2-month period bereaved persons aged 55–83 years had increased medication use, more severe illness, and worse perceived general health, the number of physician visits and hospitalizations was similar in bereaved and nonbereaved groups [29]. The authors found no sex differences in the increased risk of medication use, although women were more likely to report new and/or deteriorated health compared with men regardless of marital status. Another US study found that old-aged widowed persons with traumatic grief were less likely to use all types of healthcare services within 2 months after spouse's death compared with the bereaved subjects without traumatic grief [28]. Williams found that widowhood increased the risk of having unhealthy lifestyle behavior, developing a serious illness, and reporting worse general health only among those widowed persons who had a substantial decline in the frequency of health reminders [25]. Transition to widowhood was found to increase the first-time use of domiciliary care services among 70 years and over participants of the British Household Panel Survey [38]. However, no sex differences in the change of healthcare use associated with widowhood were reported in most studies. A recent study among Medicare beneficiaries showed substantial drop in obtaining preventive services, such as diabetic monitoring, cancer screening, and vaccination, but considerable increase in the rates of preventable hospitalization and early readmissions [26]. Like in our study, there were little gender differences in obtaining preventive services, although widowed men had substantially higher rates of preventable hospitalizations and early readmissions than widows and no long-term detrimental effects of spousal loss on individual abilities to obtain preventive healthcare services were indicated [26].

The present study adds to the previous research evidence in Denmark and other Nordic countries [39, 40] showing that all-cause and system-specific medication use was more frequent and the average daily dose for all-cause and nervous system medications was higher among women compared with same-aged men. The average daily use of respiratory medications was slightly higher among men, whereas no sex-specific pattern was observed for ATC-C medication use. 

A major strength of this study is the size, representativeness, and completeness of the Danish register data and its longitudinal nature that enabled us to examine sex differences in medication and primary healthcare use over a longer follow-up period after the event. Because the data on medication and primary healthcare use were obtained for all individuals from the 5% population sample and the twin population aged 60+ by January 1, 1996 and who became widowed in the period from 1996 to 2003, there was no room for selection bias due to nonparticipation or loss to follow-up inherent in longitudinal surveys. The present study had a sufficiently large sample size and, thus, good power to detect sex-specific pattern in health behaviors before and after spousal bereavement.

The analysis of medication use and number of GP visits over the follow-up years reflects both the effect of age on the health care utilization and the effect of conjugal bereavement. However, it is unlikely to bias the analysis of male-female differences in healthcare utilization before and after spouse's death. Clearly, our findings may be country-specific and may not be generalizable to other settings. It has been found that Sweden and Denmark have greater gender equality in the division of household labor compared with Greece or Spain [41]. Possibly, men in northern EU countries depend less on their wives for regulating health behaviors than in the more traditional southern EU. Further research should attempt to replicate this study in other populations where register data are available.

## 5. Conclusions

This study shows no sex difference in the trajectories of medication and primary healthcare use within 1 year before and up to 4 years after spouse's death. Therefore, this Danish study found no support for the hypothesis that reduced medication and primary healthcare use contribute to the explanation of more adverse health outcomes after conjugal loss in men compared with women in Denmark. 

## Figures and Tables

**Table 1:** The frequency of all-cause and system-specific medication use within 1 year before and 1 year after widowhood.

ATC	Age	Men No.	Women No.	Users before				Users after			
				Men		Women		Men		Women	
				No.	%	No.	%	No.	%	No.	%
All-cause	60–69	521	1242	416	79.8	1022	82.3	418	80.2^‡^	1081	87.0
	70–79	925	1996	771	83.4^†^	1777	89.0	807	87.2^‡^	1837	92.0
	80+	707	1030	619	87.6^†^	946	91.8	644	91.1^‡^	975	94.7

ATC-C	60–69	521	1242	166	31.9	435	35.0	204	39.2	488	39.3
	70–79	925	1996	399	43.1^†^	992	49.7	451	48.8^‡^	1098	55.0
	80+	707	1030	387	54.8^†^	629	61.1	431	61.0	653	63.4

ATC-N	60–69	521	1242	210	40.3^†^	594	47.8	253	48.6^‡^	768	61.8
	70–79	925	1996	414	44.8^†^	1204	60.3	508	54.9^‡^	1367	68.5
	80+	707	1030	399	56.4^†^	715	69.4	454	64.2^‡^	783	76.0

ATC-R	60–69	521	1242	102	19.6^†^	299	24.1	113	21.7	315	25.4
	70–79	925	1996	207	22.4^†^	526	26.4	217	23.5^‡^	566	28.4
	80+	707	1030	177	25.0	255	24.8	192	27.2	266	25.8

ATC-A	60–69	521	1242	129	24.8	338	27.2	158	30.3	353	28.4
	70–79	925	1996	284	30.7^†^	709	35.5	333	36.0	772	38.7
	80+	707	1030	292	41.3	461	44.8	342	48.4	497	48.3

ATC: Anatomical Therapeutic Chemical classification system; ATC-C: cardiovascular system; ATC-N: nervous system; ACT-R: respiratory system; ATC-A: alimentary tract and metabolism medications.

^†^ *P* value < 0.05 for sex difference in the frequency of medication use before widowhood as obtained by chi-square test.

^‡^ *P* value < 0.05 for sex difference in the frequency of medication use after widowhood as obtained by chi-square test.

**Table 2:** Average daily defined dose of all-cause and system-specific medications dispensed within 1 year before and 1 year after widowhood.

ATC	Age	Before				After				Difference^§^			
		Men		Women		Men		Women		Men		Women	
		Mean	SE	Mean	SE	Mean	SE	Mean	SE	Mean	SE	Mean	SE
All-cause	60–69	1.52	0.11	1.69	0.08	1.90	0.18	1.99	0.09	0.38	0.12	0.30	0.04
	70–79	1.98^†^	0.09	2.46	0.07	2.51^‡^	0.11	2.92	0.11	0.54	0.07	0.46	0.09
	80+	2.59	0.13	2.70	0.09	3.52	0.23	3.16	0.09	0.92*	0.18	0.46	0.06

ATC-C	60–69	0.67	0.07	0.59	0.05	0.84	0.12	0.70	0.06	0.17	0.09	0.11	0.02
	70–79	0.82^†^	0.05	0.95	0.04	1.08	0.07	1.11	0.05	0.25	0.05	0.15	0.03
	80+	1.09	0.07	1.11	0.05	1.48	0.15	1.24	0.06	0.39*	0.12	0.13	0.03

ATC-N	60–69	0.26^†^	0.03	0.40	0.02	0.34^‡^	0.04	0.52	0.03	0.08	0.02	0.13	0.01
	70–79	0.32^†^	0.02	0.55	0.02	0.43^‡^	0.03	0.70	0.03	0.12	0.02	0.15	0.02
	80+	0.44^†^	0.03	0.65	0.03	0.59^‡^	0.04	0.77	0.03	0.15	0.03	0.12	0.02

ATC-R	60–69	0.22	0.04	0.17	0.02	0.26	0.05	0.19	0.02	0.04	0.03	0.01	0.01
	70–79	0.21	0.03	0.21	0.02	0.25	0.03	0.24	0.02	0.04	0.01	0.02	0.01
	80+	0.17^†^	0.02	0.10	0.01	0.20^‡^	0.02	0.12	0.01	0.02	0.01	0.02	0.01

ATC-A	60–69	0.12	0.02	0.15	0.01	0.15	0.02	0.17	0.01	0.03	0.02	0.02	0.01
	70–79	0.18	0.02	0.21	0.01	0.23	0.02	0.27	0.03	0.04	0.01	0.06	0.03
	80+	0.27	0.03	0.29	0.02	0.41	0.04	0.36	0.02	0.14*	0.03	0.07	0.01

ATC: Anatomical Therapeutic Chemical classification system; ATC-C: cardiovascular system; ATC-N: nervous system; ACT-R: respiratory system; ATC-A: alimentary tract and metabolism medications; DDD: daily defined dose.

*P value < 0.05 for sex differences in the change of medication use before and after widowhood as obtained by two-sided *t*-test.

^†^ *P* value < 0.05 for sex difference in the average DDD before widowhood as obtained by two-sided *t*-test.

^‡^ *P* value < 0.05 for sex difference in the average DDD after widowhood as obtained by two-sided *t*-test.

^§^Difference = average DDD after, average DDD before.
